# Programmed Cell Death of Endothelial Cells in Ischemic Heart Disease: Mechanism and Potential Cell and Gene Therapeutic Prospects

**DOI:** 10.3390/bioengineering13060661

**Published:** 2026-06-04

**Authors:** Zijia Sun, Lei Chen, Yingying Cao, Bingyang Dai, Lintao Wang, Ling Zhang

**Affiliations:** 1 Department of Rehabilitation, Children’s Hospital of Nanjing Medical University, Nanjing 210003, China; szjele@163.com; 2 Department of Cardiology, The Affiliated Hospital of Xuzhou Medical University, Xuzhou 221002, China; drleichen@163.com; 3 National Heart Centre Singapore, Singapore 169609, Singapore; 4 Zhejiang Cancer Hospital, Hangzhou Institute of Medicine (HIM), Chinese Academy of Sciences, Hangzhou 310022, China; caoyy@zjcc.org.cn; 5 Department of Biomedical Engineering, Faculty of Engineering, The Hong Kong Polytechnic University, Hong Kong SAR, China; 6 Shenzhen Research Institute, The Hong Kong Polytechnic University, Shenzhen 518000, China; 7 Department of Cardiology, Nanjing Drum Tower Hospital, Affiliated Hospital of Medical School, Nanjing University, Nanjing 210003, China

**Keywords:** ischemic heart disease, programmed cell death, endothelial cells, cell therapy, gene therapy

## Abstract

Ischemic heart disease (IHD) is the leading cause of death worldwide, accounting for over eight million deaths each year. IHD encompasses a spectrum of conditions, including atherosclerosis (AS), myocardial infarction (MI), and ischemia/reperfusion (I/R) injury. Programmed cell death (PCD) of endothelial cells (ECs) plays a critical role in IHD pathogenesis, causing microvascular dysfunction, barrier disruption and exacerbation of cardiac injury. PCD involves different signaling pathways, but they are interconnected. Therefore, it is crucial to understand the mechanisms underlying the various forms of PCD in ECs to develop therapeutic strategies for IHD. This review focuses on the molecular mechanisms of PCD of ECs in IHD and provides comprehensive summary of potential cell and gene therapy therapeutic strategies for the treatment of IHD.

## 1. Introduction

Ischemic heart disease (IHD) is the leading global cause of disability and death, imposing a substantial disease burden [1]. Atherosclerosis (AS) is the pathological basis of IHD, and the progression of atherosclerotic plaque formation to acute myocardial infarction (MI) and subsequent lethal myocardial ischemia/reperfusion (I/R) injury constitutes a continuous pathological chain. Endothelial dysfunction serves as both the initiating factor and critical component in AS development. The programmed cell death (PCD) of endothelial cells (ECs) has been demonstrated to be closely associated with pathophysiological processes of IHD, including plaque destabilization and rupture, microvascular dysfunction, and inflammatory cell infiltration [2]. It has been established that multiple PCD pathways in ECs collectively drive endothelial injury and disease progression. Therefore, understanding the PCD of ECs in IHD is essential. In recent years, cell and gene therapy have shown therapeutic potential for protecting the PCD and function of ECs in IHD, which provide novel approaches for the treatment of IHD and hold transformative prospects for clinical application. This review outlines mechanisms of key PCD pathways of ECs in IHD and summarizes potential therapeutic strategies. Emphasis is placed on cell and gene therapy, serving as a reference for promoting clinical translation research in IHD.

Our search strategy involved querying major databases (e.g., PubMed) using terms such as “endothelial cell programmed cell death”, “cell and gene therapy”, “atherosclerosis”, “myocardial infarction”, “myocardial I/R injury”, and so on. Inclusion criteria required that studies report on the impact of endothelial cell programmed cell death on ischemic heart disease (IHD), the mechanisms underlying programmed cell death in endothelial cells (ECs), potential therapeutic strategies, and cell or gene therapy approaches that protect ECs. Irrelevant topics, poor-quality studies, and articles for which the full text could not be obtained were excluded. Numerous studies discussing natural agents that protect against endothelial programmed cell death were also excluded because of their unclear pharmacological mechanisms. This approach ensured a focused, representative selection of key studies.

## 2. The Molecular Mechanism of PCD of ECs

ECs—the primary cells lining blood vessels—maintain vascular homeostasis, regulate permeability, and preserve barrier integrity. Vascular ECs form a selective barrier that regulates the blood–tissue exchange of substances and cells, maintaining tissue microenvironmental stability and function [3]. Dysfunction of ECs compromises vascular wall integrity and contributes directly to the pathogenesis of IHD [4]. As is well known, PCD of ECs is a primary factor contributing to endothelial cell dysfunction, including apoptosis, pyroptosis, necroptosis, autophagy, ferroptosis, cuproptosis, PANoptosis, parthanatos, and so on [5,6,7,8]. A comprehensive understanding of PCD of ECs in IHD is essential for uncovering mechanisms and exploring therapeutic targets [9].

### 2.1. Apoptosis of ECs

**Figure 1:**
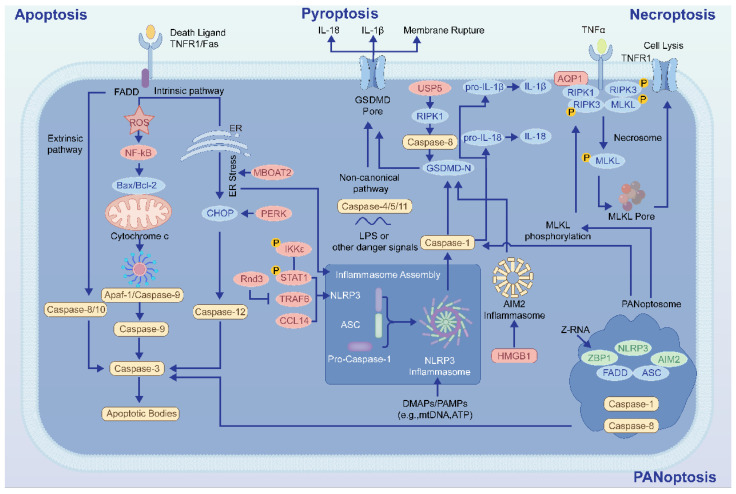
Overview of endothelial cell apoptosis, pyroptosis, necroptosis and PANoptosis signaling pathways in IHD. (1) The intrinsic pathway of apoptosis can be activated by stimuli such as growth factor deprivation, DNA damage and oxidative stress. Pro-apoptotic proteins within Bax are activated, leading to increased permeability of the mitochondrial outer membrane. Cytochrome c is released into the cytoplasm, initiating the caspase cascade. During endoplasmic reticulum stress, upregulated PERK can activate CHOP, thereby inducing the activation of Caspase-12 and apoptosis. The extrinsic pathway is directly initiated by death ligands (FasL, TNF-α) binding to cell membrane death receptors. (2) Pyroptosis relies on gasdermin family proteins as core effector molecules. The classical pathway is mediated by Caspase-1 activation, while the non-classical pathway is mediated by Caspase-4/-5 in humans and Caspase-11 in mice. (3) Following death receptor activation, RIPK1 and RIPK3 form necrosomes via their RHIM domains. RIPK3 becomes phosphorylated and activates MLKL. Activated MLKL oligomerizes and translocates to the plasma membrane, disrupting membrane integrity and causing cell lysis. Aquaporin 1 (AQP1) inhibits RIPK3 and MLKL expression by binding to RIPK1, exerting an anti-necroptosis effect. (4) Upon stimulation by pathogens, injury signals, or cytokine storms, innate immune sensors (ZBP1, AIM2, NLRP12, etc.) assemble into the PANoptosome multiprotein complex, simultaneously converging core molecular components of pyroptosis, apoptosis, and necroptosis. This complex coordinates the activation of the caspase family and RIPK kinases, ultimately executing a cell death program that concurrently exhibits characteristics of all three types of cell death. In this figure, arrows (→) represent promotion or activation, and blunt-ended lines (—|) represent inhibition or suppression.

ECs undergo apoptosis via classical pathways, intrinsic (mitochondrial/ER) and extrinsic (death receptor). Both intrinsic and extrinsic pathways activate the caspase family through different mechanisms, ultimately leading to PCD [10]. The mitochondrial pathway of apoptosis signaling represents the primary apoptotic mechanism triggered by intracellular stress signals. Oxidative stress, DNA damage, or metabolic disturbances increase mitochondrial membrane permeability, which promotes the release of cytochrome C into cytoplasm and the formation of the apoptosome (comprising cytochrome C, apoptotic protease-activating factor-1 (Apaf-1), and adenosine triphosphate (ATP)). This assembly activates caspase-9, triggering a cascade that culminates in effector caspase activation (e.g., caspase-3), ultimately leading to apoptosis [11,12]. Moreover, the mitochondrial pathway has been shown to be closely associated with oxidative stress in ECs. The nuclear factor erythroid 2-related factor 2 (Nrf2)/heme oxygenase-1 (HO-1) signaling pathway has been identified as a core mechanism of cellular antioxidant defense. Its activation could mitigate mitochondrial damage, suppress excessive reactive oxygen species (ROS), and attenuate apoptosis [13]. Various aspects of natural agents exert protective effects against EC injury by activating Nrf2/HO-1 signaling [13]. However, due to the unclear pharmacological mechanism and the lack of detailed preclinical and clinical studies, we may not provide further discussion on these drugs and the development of new drugs for the treatment of IHD. Consequently, the mitochondrial pathway functions not only as the execution route of apoptosis but also as a pivotal regulatory point in cellular stress responses. On the other hand, the endoplasmic reticulum (ER) pathway of apoptosis signaling is initiated upon excessive or prolonged ER stress, causing accumulation of unfolded/misfolded proteins and dysregulated calcium. This, in turn, exacerbates the ER burden and ultimately induces apoptosis [14,15]. In addition, the death receptor pathway, an extrinsic classical pathway, is initiated through ligands binding to cell-surface death receptors, including Fas (CD95) and the tumor necrosis factor receptor (TNFR) [16]. Upon receptor–ligand binding, the death-inducing signaling complex (DISC) forms, recruiting FADD to activate caspase-8. Subsequently, it activates the effector caspase-3 or promotes the mitochondrial pathway by cleaving the Bid protein, inducing apoptosis (Figure 1) [17,18].

### 2.2. Pyroptosis of ECs

Endothelial cell pyroptosis involves inflammasome activation and gasdermin family cleavage, causing membrane pore formation and pro-inflammatory cytokine release [19]. At present, pyroptosis is classified into three pathways, the caspase-1-gasdermin-dependent classical pathway, the caspase-4/5/11-gasdermin-dependent non-classical pathway, and other pathways. The classical pathway is primarily inflammasome-mediated, which consists of the NOD-like receptor (NLR), the adaptor ASC, and caspase-1 [20,21]. Activation of caspase-1 cleaves GSDMD and pro-IL-1β/18 and then the GSDMD-N-terminal fragment (GSDMD-NT) forms plasma membrane pores, enabling IL-1β and IL-18 release [22,23]. In the non-canonical pathway, intracellular lipopolysaccharide (LPS) directly activates caspase-4/5/11, triggering GSDMD cleavage—essential for pore formation and pyroptosis (Figure 1) [24].

### 2.3. Necroptosis of ECs

Necrosis is mediated by the receptor-interacting protein kinase 1 (RIPK1)–RIPK3 mixed-lineage kinase domain-like protein (MLKL) signaling pathway, typically triggered by cellular damage or inflammatory stimuli. This leads to the activation of RIPK1, which then interacts with RIPK3 to form the “necrosome” complex. Subsequently, RIPK3 phosphorylates and activates MLKL, which disrupts the plasma membrane, causing cell swelling, rupture, and necrotic death [25,26]. The cascade of the RIPK1/RIPK3/MLKL pathway serves as both the execution mechanism for cell death and a critical trigger for inflammatory responses. When ECs undergo necroptosis via this pathway, they release pro-inflammatory cytokines and DAMPs, amplifying local inflammation and driving pathological progression [27,28]. Furthermore, necroptosis is tightly intertwined with multiple inflammation-related pathways. For instance, TNF-α activates RIPK1 via TNF receptors in ECs, triggering RIPK3 and MLKL phosphorylation—leading to necroptosis and amplified inflammation (Figure 1) [29].

### 2.4. PANoptosis of ECs

PANoptosis is a programmed inflammatory cell death pathway combining pyroptosis, apoptosis, and necroptosis, and is closely linked to IHD [30]. Endothelial PANoptosis is regulated by the PANoptosome complex, whose formation is essential for crosstalk among multiple pathways [31]. The core structure of PANoptosome comprises sensors including Z-DNA binding protein 1 (ZBP1), the Nod-like receptor protein (NLRP) family, absent in melanoma 2 (AIM2), adapter proteins (including FADD, ASC, effector including MLKL), and the caspase protein family [32]. PANoptosis is initiated when sensor proteins detect danger signals, triggering PANoptosome assembly via adapter proteins. These adapters act as molecular scaffolds that coordinate and activate key molecules across PCD pathways (Figure 1) [33].

### 2.5. Ferroptosis of ECs

**Figure 2:**
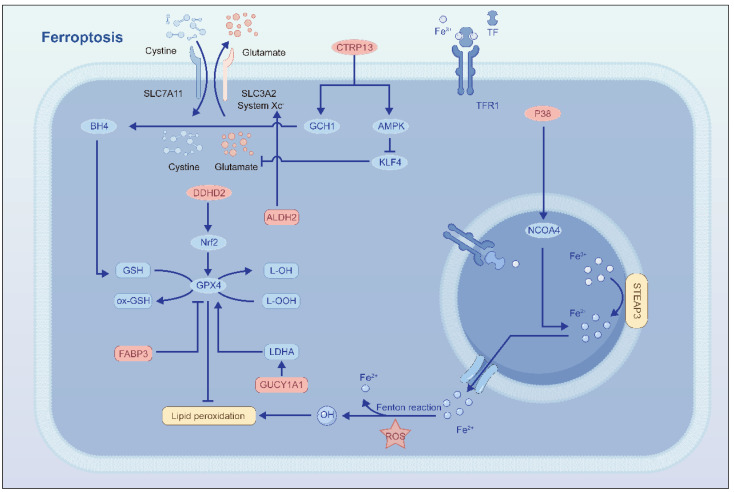
Overview of endothelial cell ferroptosis signaling pathways in IHD. Dysfunction of the cystine/glutamate antiporter (System Xc^−^, composed of SLC7A11) reduces glutathione (GSH) synthesis, impairing the activity of phospholipid hydroperoxidase GPX4 and its ability to detoxify membrane lipid peroxides—a central execution step of ferroptosis. FABP3, DDHD2, and GUCY1A1 regulate ferroptosis in endothelial cells by modulating GPX4 protein expression. C1q/tumor necrosis factor-related protein 13 (CTRP13) not only regulates GPX4 expression via the GCH1/BH_4_ signaling pathway but also suppresses ferroptosis by activating AMPK and regulating System Xc^−^. Activation of the aldehyde dehydrogenase 2 (ALDH2) gene pathway also regulates endothelial ferroptosis via System Xc^−^. Additionally, p38 induces mitochondrial ferritin autophagy and ferroptosis by activating the nuclear coactivator 4 (NCOA4) pathway. In this figure, arrows (→) represent promotion or activation, and blunt-ended lines (—|) represent inhibition or suppression.

Ferroptosis is an iron-dependent form of PCD driven by lipid peroxide accumulation from iron overload, causing membrane damage and cell death [34]. Growing evidence highlights ferroptosis’s pivotal role in ECs dysfunction [35,36]. Ferroptosis is triggered by the iron-catalyzed Fenton reaction, generating large amounts of ROS—especially lipid peroxides. Iron ions disrupt membrane integrity by catalyzing the peroxidation of polyunsaturated fatty acid (PUFA) phospholipid peroxidation, leading to cellular dysfunction and death [37]. Abnormally elevated intracellular iron levels in ECs, such as increased transferrin receptor 1 (TfR1) expression, promote iron influx and increase iron load, further exacerbating lipid peroxidation [38,39]. Lipid peroxidation damages both cellular and mitochondrial membranes, reducing membrane potential, impairing mitochondrial function, and promoting cell death [40]. Glutathione peroxidase 4 (GPX4) is a key enzyme that suppresses ferroptosis by reducing lipid peroxides to preserve membrane lipid integrity. Its downregulation or loss of function triggers ferroptosis (Figure 2) [41].

### 2.6. Autophagy of ECs

**Figure 3:**
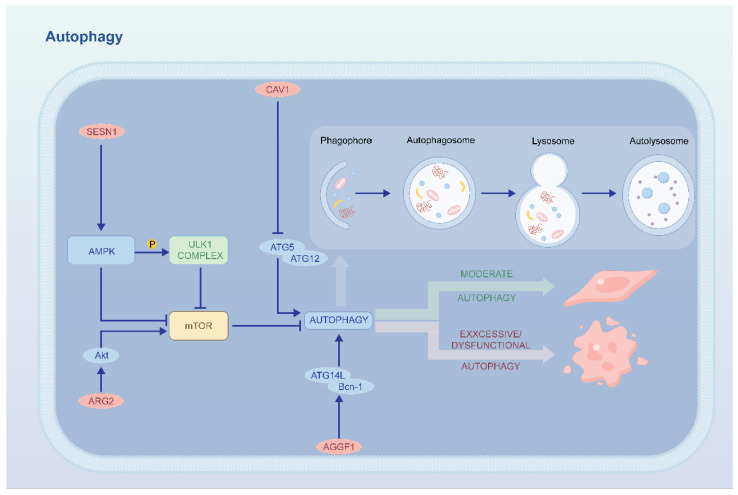
Overview of endothelial cell autophagy signaling pathways in IHD. The AMPK/mTOR pathway is activated under various stress conditions such as oxidative stress and nutrient deprivation. AMPK plays a central role in maintaining cellular homeostasis by inhibiting mTOR signaling, promoting autophagy, and regulating lipid metabolism and glucose utilization. The mTOR pathway, conversely, primarily mediates cell growth, anabolic processes, and inflammatory activation. The balance between these two pathways determines whether cells undergo repair or damage. Sestrin 1 (SESN1), as an upstream activator of AMPK, exhibits expression levels positively correlated with autophagy activity. Arginase 2 (ARG2) enhances mTORC1 activity, thereby suppressing autophagy. Caveolin-1 influences autophagosome formation by interacting with the ATG5-ATG12 complex; its absence increases autophagic flux. Angiogenic factor 1 (AGGF1) participates in regulating endothelial autophagy. In this figure, arrows (→) represent promotion or activation, and blunt-ended lines (—|) represent inhibition or suppression.

Autophagy-dependent cell death results from excessive or dysregulated autophagy. While autophagy protects ECs against oxidative stress and inflammation—thereby preserving vascular function—its overactivation can trigger this form of cell death, leading to IHD. Autophagy is classified by its cargo and delivery mechanisms. The three predominant classifications are macroautophagy, microautophagy, and chaperone-mediated autophagy (CMA). Among these, macroautophagy is the most prevalent [42]. Autophagy-related pathways encompass the mammalian target of the rapamycin (mTOR)-dependent pathway and the mTOR-independent pathway [43]. In response to stress or stimulation, the mammalian target of rapamycin complex 1 (mTORC1) is inactivated, activating the UNC-51-like autophagy receptor kinase 1 (ULK1) complex to initiate phagophore assembly. Upon autophagy induction, ULK1 phosphorylates the class III phosphatidylinositol 3-kinase (PI3K) complex I, generating phosphatidylinositol-3-phosphate (PI3P) and promoting autophagosome membrane nucleation [44]. Subsequently, two ubiquitin-like conjugation systems are engaged to promote autophagosomal membrane elongation: the ATG5-ATG12-ATG16L complex and microtubule-associated protein 1 light chain 3-phosphatidylethanolamine (LC3-PE) [45]. In mTOR-independent autophagy pathways, Ca^2+^, AMP-activated protein kinase (AMPK), mitogen-activated protein kinase (MAPK)/c-Jun N-terminal kinase (JNK), and microRNAs (miRNAs) have been identified as key regulators of autophagy (Figure 3) [43].

## 3. PCD of ECs Plays a Pivotal Role in the Development and Progression of IHD

### 3.1. Programmed Cell Death of ECs in AS

Atherosclerosis is a chronic arterial wall inflammation causing coronary heart disease. In the early stage of vascular lesions, lipid deposition occurs in the intima first, followed by inflammatory cell infiltration, fibrous tissue hyperplasia, and eventually plaque formation [46,47]. Unstable plaque rupture and subsequent thrombosis can cause IHD [48]. Risk factors such as hypertension, hyperlipidemia and diabetes directly cause vascular damage by altering the phenotype of EC at arterial bifurcations [49,50]. ECs lining blood vessels are not uniform; they vary in structure, function, and gene expression depending on their location. In atherosclerosis-prone regions, disturbed blood flow alters endothelial behavior. These cells show increased expression of inflammatory mediators (e.g., VCAM-1, MCP-1), reduced nitric oxide production, and a procoagulant shift. In contrast, flow-protected areas maintain normal alignment and anti-inflammatory profiles. Importantly, endothelial phenotypes are shaped by both fixed (epigenetic) and dynamic (microenvironmental) signals [51]. This plasticity allows the endothelium to adapt but also makes it vulnerable to modern risk factors. This heterogeneity is exactly why atherosclerosis (AS) arises at specific, focal locations. Both animal and human studies show a complex, close interplay between endothelial cells (ECs) and mononuclear phagocytes as atherosclerosis (AS) progresses.

Oxidation of low-density lipoprotein (LDL) generates oxidized LDL (ox-LDL), which is taken up by ECs and macrophages via scavenger receptors, driving foam cell formation and being an initial step in atherosclerotic lesion development [52,53]. On one hand, risk factors drive ECs to release growth factors and cytokines. This increases vascular permeability and alters the underlying extracellular matrix, which in turn triggers mononuclear phagocyte infiltration and aggregation, fueling AS development [54,55]. On the other hand, mononuclear phagocytes that have gathered in the damaged vessel wall use scavenger receptors to take up lipid particles and turn into foam cells. These foam cells then secrete growth factors and cytokines such as tumor necrosis factor α (TNF α) and interleukin 1β (IL 1β), further damaging ECs and worsening their dysfunction. This creates a vicious cycle [56,57]. Damaged ECs also allow vascular smooth muscle cells (VSMCs) to migrate into the subendothelial space, where they promote plaque formation [58,59]. At the same time, transforming growth factor β (TGFβ) released by VSMCs can trigger endothelial mesenchymal transition (EndMT) in ECs. ECs undergoing EndMT lose their barrier function and take on pro-inflammatory and pro-fibrotic traits, which accelerates AS progression [60]. These findings indicate that EC dysfunction plays a pivotal role in AS initiation and progression, with programmed EC death being a key contributing factor.

#### 3.1.1. Apoptosis of ECs in AS

In AS, the apoptosis of ECs is mainly achieved through the extrinsic death receptor pathway and the intrinsic mitochondrial pathway [61]. Early in atherosclerosis, EC apoptosis promotes lipid deposition and inflammatory cell infiltration, then drives plaque formation [62,63]. In addition, apoptotic ECs may undergo secondary necrosis, releasing pro-inflammatory mediators and cellular debris that intensify local inflammation and expand the plaque necrotic core, which is a hallmark of advanced AS [64].

In the initiation and progression of AS, EC apoptosis serves as a critical pathological event, with its molecular mechanisms primarily revolving around the imbalance between pro-apoptotic proteins (e.g., BAX, caspase) and anti-apoptotic proteins (e.g., BCL-2). Oxidative stress activates the IKKβ/IκBα/NF-κB pathway, and upregulates the Bax/Bcl-2 ratio and cleaved caspase-3 expression, thereby inducing apoptosis in human aortic endothelial cells (HAECs) (Figure 1) [65]. In contrast, Poly(I:C) exerts anti-apoptotic effects in a mouse model of atherosclerosis. In vitro studies have confirmed that the possible mechanism is the upregulation of BCL-2 and downregulation of BAX and caspase-3/9 through JAK/STAT activation [66]. Furthermore, both glutathione and Akebia saponin D (ASD) protect ECs by restoring the Bax/Bcl-2 balance and inhibiting caspase activation [67,68].

ER stress constitutes an additional significant pro-apoptotic mechanism. In human umbilical vascular endothelial cells (HUVECs) that had been stimulated with LDL, ox-LDL upregulates ER stress marker proteins, including glucose-regulated protein 78 (GRP78), p-PERK, p-IRE1α, and C/EBP homologous protein (CHOP). This upregulation results in the activation of the PERK/eIF2α/CHOP and IRE1α/sXBP1 signaling axes. This leads to EC apoptosis (Table 1) [69]. Statins, such as rosuvastatin, significantly reduce EC apoptosis and vascular dysfunction by dual inhibition of the PERK/eIF2α/CHOP and IRE1α/sXBP1 pathways in ApoE^−^/^−^ mice (Figure 1) [69]. Statins exert multiple effects on atherosclerosis and rosuvastatin can slow the progression of atherosclerotic cerebral infarction (ACI) in patients, partly through inhibiting OX40 ligand expression and upregulating peroxisome proliferator-activated receptor gamma (PPAR-γ) in endothelial cells [70]. Inhibition of endoplasmic reticulum (ER) stress may represent another important protective mechanism of rosuvastatin in the aortic endothelium. However, further studies are needed to confirm these findings. Oxidative stress, as the core initiating factor driving apoptosis of ECs, activates the aforementioned apoptotic pathways but also induces inflammation [71]. Nrf2 activation has been demonstrated to enhance cellular antioxidant capacity and to suppress the expression of adhesion molecules (VCAM-1 and ICAM-1), thereby delaying the onset of inflammation in AS [72]. In the postmenopausal phase, estrogen plays a crucial role in maintaining endothelial homeostasis by suppressing oxidative stress and inflammation through the activation of ERα/ERβ receptors [73,74]. However, the decline in estrogen levels after menopause, in conjunction with the prevalence of hyperhomocysteinemia, collectively exacerbates apoptosis of ECs and vascular damage [75].

In summary, EC apoptosis in AS progression manifests as a molecular pattern characterized by upregulation of BAX/caspase-3/9 and downregulation of BCL-2. This process is controlled by multiple pathways, including NF-κB, ER stress, JAK/STAT, and so on. It is closely associated with oxidative stress, inflammation, and specific hormonal environments (such as postmenopausal status).

#### 3.1.2. Pyroptosis of ECs in AS

Elevated ox-LDL, disturbed blood flow, metabolic abnormalities, and toxic substances are key risk factors inducing pyroptosis of ECs in AS. Pyroptotic ECs undergo physiological changes that trigger the release of inflammatory factors, promoting vascular inflammation and the death of macrophages, VSMCs, and arterial ECs. This exacerbates necrotic core formation, increases atherosclerotic plaque instability, and elevates thrombosis risk [94,95]. In early AS, ox-LDL and disturbed blood flow triggers mild, scattered pyroptosis in ECs at vulnerable arterial sites, initiating disease progression [61,96]. Upon uptake by ECs, ox-LDL causes lysosomal damage and robust ROS production, where disturbed blood flow enhances ROS generation, and then activates the NLRP3 inflammasome-dependent GSDMD cleavage and leads to the formation of pores on ECs’ membranes [97]. Besides NLRP3, AIM2 inflammasomes can also activate pyroptosis (Figure 1) [98]. In advanced AS, pyroptosis of ECs in the fibrous cap disrupts endothelial continuity, exposing procoagulant substances and promoting platelet adhesion and thrombus formation, finally leading to vascular occlusion [99]. Low-shear stress was found to trigger endothelial pyroptosis along with IκB kinase ε (IKKε) phosphorylation. In ApoE^−^/^−^ mice consuming a high-cholesterol diet, IKKε silencing markedly diminished aortic arch atherosclerotic lesions and concurrently suppressed low-shear stress-driven endothelial pyroptosis and NLRP3 expression. Mechanistically, IKKε upregulated NLRP3 by stimulating signal transducer and activator of transcription 1 (STAT1), which subsequently bound to the NLRP3 promoter in in vitro studies (Figure 1) [77]. TMAO, an independent risk factor for AS, drives pyroptosis by upregulating membrane bound O-acyltransferase domain containing 2 (MBOAT2) in vivo and vitro, inducing ER stress and mitochondrial ROS production, or by activating NLRP3 through ROS-mediated succinate dehydrogenase complex iron sulfur subunit B (SDHB) upregulation (Figure 1) [76].

Beyond direct risk factor stimulation, multiple signaling pathways regulate the NLRP3 inflammasome, influencing pyroptosis of ECs and AS progression. In AS, PI3K/AKT activation suppresses NLRP3 inflammasome partly by inhibiting NF-κB, which directly upregulates NLRP3 expression and exacerbates the pyroptosis of ECs [100]. Furthermore, reduced expression of Rho family GTPase 3 (Rnd3) has been demonstrated to exacerbate the progression of AS in ApoE^−^/^−^ mice [78]. It binds to TNF receptor associated factor 6 (TRAF6), facilitating K48-dependent ubiquitination and degradation, resulting in the blockage of NF-κB and mitigating the pyroptosis of ECs (Figure 1). TRAF6 knockdown countered Rnd3 knockout-evoked exacerbation of EC pyroptosis in vivo and vitro [78]. Moreover, the chemokine CCL14 promotes M1 and endothelial pyroptosis of ECs by activating the NF-κB/NLRP3 axis (Figure 1) (Table 1) [79]. In summary, pyroptosis of ECs drives vascular inflammation in AS via a complex signaling network. These pathways exhibit a high degree of complexity and interconnectedness.

#### 3.1.3. Necroptosis of ECs in AS

In AS, ox-LDL has been demonstrated to upregulate the expression of RIPK1, RIPK3, and MLKL, thereby inducing necroptosis of ECs. Aquaporin-1 (AQP1), mainly enriched in ECs both in expression and spatial location, exerts anti-necroptotic effects by binding with RIPK1 and inhibiting the expression of RIPK3 and MLKL to increase plaque stability in a mouse model (Figure 1) [82]. However, RIPK3 knockout in ECs has been shown to promote AS progression, suggesting RIPK3 has necroptosis-independent protective roles in ECs [101].

Modulation of the necroptosis pathway has emerged as a novel therapeutic strategy for AS. Necrostatin-1, a small molecular alkaloid, was identified as an inhibitor of necroptosis. Emerging evidence suggests that Necrostatin-1 possesses numerous pharmacological activities, including anti-cancer activity, protective effects on heart, and so on [102]. In ox-LDL stimulated ECs, Necrostatin-1 ameliorates ox-LDL induced NO reduction and vascular adhesion molecules including VCAM-1 and E-selectin. Further research has demonstrated that Necrostatin-1 inactivates RIPK1 and suppresses NF-κB P65 nuclear translocation, suggesting a potential therapeutic strategy for AS, but further in vivo experimental validation is still required [103]. Cilostazol alleviates vascular inflammation and delays atherosclerosis progression by inhibiting necroptosis of ECs and IL-1β production [104]. The pharmaceutical agent Nattokinase has been demonstrated to exert beneficial therapeutic effects by impeding necroptosis through a multitude of mechanisms. These findings suggest that targeting factors are associated with the necroptosis pathway.

#### 3.1.4. PANoptosis of ECs in AS

Research has demonstrated that the AIM2 PANoptosome can promote the formation of an inflammatory microenvironment by inducing PANoptosis, thereby accelerating AS progression [105]. This PANoptosome recognizes oxidative DNA damage and replication stress, which can exacerbate vascular endothelial injury [106]. Furthermore, the NLRP3 inflammasome is aberrantly activated in AS, where it senses cholesterol crystals and ox-LDL to drive pyroptosis and cooperatively induce apoptosis and necroptosis [20]. In diabetes, hyperglycemia triggers sensor proteins (e.g., ZBP1) via oxidative stress and mitochondrial dysfunction, leading to PANoptosome assembly and subsequent EC death through multi-pathway crosstalk [107]. This distinctive “hybrid cell death” may amplify endothelial barrier disruption, accelerating atherosclerotic lesion formation. Bioinformatics analysis identified four key PANoptosis-related genes including ZBP1 and AIM2 significantly associated with AS (Figure 1) [108]. Therefore, multi-target drugs or combination therapies are necessary to avoid compensatory activation of other death subroutines. However, further exploration is necessary to elucidate the specific molecular mechanisms, particularly the assembly and regulation of PANoptosomes in ECs.

#### 3.1.5. Ferroptosis of ECs in AS

Iron overload, lipid peroxidation, and ROS generation are correlated in ECs of diabetic mice, contributing to vascular dysfunction, which in turn promotes the onset and progression of AS [109]. Chronic iron overload exacerbates atherosclerosis in ApoE^−^/^−^ mice by increasing nitrotyrosine, promoting endothelial dysfunction and plaque instability [110]. Iron overload in ApoE^−^/^−^ mice also increases prostaglandin production via upregulation of COX-2 in aortic ECs, causing oxidative imbalance and exacerbating atherosclerosis [111]. Iron chelators such as deferoxamine (DFO) and deferiprone bind free iron, inhibiting its participation in redox reactions and the Fenton reaction, thereby suppressing ferroptosis in ECs [112]. However, high-dose DFO often associates with elevated pulmonary blood pressure, exacerbates Vibrio and Yersinia infections, and causes visual and auditory neurotoxicity. Although most toxic side effects are reversed upon drug discontinuation, further evaluation of its safety is still needed [112]. Ferrostatin-1 (Fer-1), the first reported ferroptosis inhibitor (2012), has been extensively used as a reference compound in the past decade. Fer-1 is an arylalkylamine that prevents lipid hydroperoxide accumulation in an erastin-mediated ferroptosis model in HT-1080 cells [113]. It has been shown to suppress lipid peroxidation in vivo by scavenging ROS, reducing lipid peroxide accumulation, and inhibiting ferroptosis in ECs, thereby alleviating AS in high-fat-diet ApoE^−^/^−^ mice. Further in vitro experiments demonstrated that it upregulates the levels of SLC7A11 and GPX4, which may be the primary molecular mechanism [114,115].

It has been shown that environmental pollutants (e.g., PM2.5) transmit miR-3529-3p via alveolar macrophage-derived extracellular vesicles, which targets and inhibits endothelial cell ferritin heavy chain (FTH1), disrupting iron homeostasis [116]. Fer-1 and DFOM inhibit PM2.5-induced AS progression by regulating ferroptosis of ECs [117]. A lower plaque burden and increase in endothelial cells with decreased lipid peroxidation was found in a radiation-associated atherosclerosis (RAA) mouse model treated with ferroptosis inhibitors. Further experiments confirmed that radiation induced the occurrence of ferroptosis in HAECs. In vitro study confirmed that radiation triggers mitochondrial ferritinophagy and ferroptosis via the p38/NCOA4 and domain-containing protein 2(DDHD2)-mediated Nrf2/GPX4 pathway (Figure 2) (Table 1) [85,89]. Another study demonstrated that ionizing radiation (IR) accelerated the progression of atherosclerotic plaque by regulating EC ferritin phagocytosis/ferritin deposition in a P38/NCOA4-dependent manner. This reveals that radiation may affect AS through different mechanisms of ferroptosis, and more related pathway research is still needed [85,89]. Furthermore, specific metabolites modulate VEC ferroptosis via distinct pathways: (1) Hcy acts through the system Xc^−^–GSH–GPX4 axis; (2) PGPC downregulated GPX4 by activating FABP3 via CD36; and (3) Neu5Ac triggers ferroptosis via SLC3A2, leading to endothelial dysfunction and promoting atherosclerosis [41,87,118].

During AS pathogenesis, multiple signaling pathways regulate the ferroptosis of ECs. The Nrf2 pathway is a key antioxidant pathway that upregulates GPX4 (a critical ferroptosis inhibitor) and SLC7A11 (the cystine/glutamate antiporter light chain), thereby suppressing lipid peroxidation, iron accumulation, ferroptosis of ECs, and AS progression [119]. Magnesium ions enhance SLC7A11 expression by activating the MAPK pathway, thereby inhibiting EC ferroptosis [120]. The GTP cyclohydrolase I/tetrahydrobiopterin (GCH1/BH4) axis is a key antioxidant pathway that protects ECs from ferroptosis by mitigating mitochondrial oxidative stress. C1q/TNF-related protein 13 (CTRP13) activates this axis, improves mitochondrial function, and reduces oxidative stress, thereby inhibiting the ferroptosis of ECs and slowing AS progression in high-fat-diet-induced ApoE^−^/^−^ mice [83]. CTRP13 also activates AMPK to suppress KLF4 expression, enhance metabolic stress adaptation, upregulate antioxidant enzymes, and alleviate oxidative stress and mitochondrial dysfunction—thereby inhibiting ferroptosis (Figure 2) (Table 1) [84]. This means that CTRP13 may be the key target of treating atherosclerosis by inhibiting ferroptosis. Moreover, the GCH1/BH4, PI3K/AKT/mTOR, and RAP1B/NRF2 pathways are also key regulators of ferroptosis of ECs [83,121,122]. In summary, ferroptosis impairs endothelial function and vascular homeostasis via iron dyshomeostasis and lipid peroxidation. These studies explore ferroptosis inhibition as a therapeutic target for AS.

#### 3.1.6. Autophagy of ECs in AS

In early AS, autophagy is induced in cells exposed to oxidative stress and metabolic distress, protecting them from damage. Under mild oxidative stress, autophagy promotes cellular recovery by clearing damaged components—exerting a key anti-atherosclerotic effect. However, AS preferentially develops at arterial bifurcations and curvatures, where disturbed or low shear stress impairs autophagy. This impairment accelerates EC inflammation, apoptosis, and senescence, thereby promoting lesion formation [123]. In high-shear-stress (atheroprotective) regions, endothelial-specific ATG5 deletion promotes significant atherosclerotic plaque formation [124], but shows that shear stress levels differentially regulate autophagy. For instance, oscillatory shear stress (OSS)-activated integrin β3 in ECs impairs autophagy flux, causing endothelial dysfunction and AS [125]. Under LSS, mTOR activation inhibits AMPK and impairs autophagy in ECs. Conversely, high shear stress promotes effective autophagy in ECs, suppressing atherosclerotic plaque development by reducing apoptosis, senescence, and inflammation [124]. Ox-LDL-induced endothelial injury disrupts the AMPK/mTOR pathway, reducing AMPK phosphorylation and increasing mTOR phosphorylation, leading to impaired autophagy, lipid accumulation, and inflammation [90].

Sestrin 1 (SESN1), an upstream AMPK activator, positively correlates with autophagy activity and silencing SESN1 abolishes orientin-induced AMPK activation in HUVECs induced by ox-LDL (Figure 3) [90]. Similarly, arginase 2 (ARG2) suppresses AMPK signaling and impairs endothelial autophagy in advanced atherosclerosis by activating the RICTOR–mTORC2–AKT cascade to enhance mTORC1 activity in vitro and in vivo (Table 1) [91]. C1q/tumor necrosis factor-related protein 9 (CTRP9) restores PA-inhibited autophagic flux via AMPK activation, thereby alleviating PA-induced EC senescence, highlighting the critical role of AMPK in maintaining EC homeostasis (Figure 3) [126]. It was found that Caveolin-1 deficiency significantly reduces vascular inflammatory responses and atherosclerotic lesions in an atherosclerotic mouse model. The potential mechanism is that Cav-1 interacted with the ATG5-ATG12 complex and directed autophagosome components to lipid rafts, a process that regulated autophagosome formation and autophagic flux (Figure 3) [92]. In summary, the autophagy of ECs in AS progression hinges on a dynamic, tightly regulated balance. Under physiological or mild stress, it acts as a key quality-control mechanism—preserving endothelial barrier function and anti-atherosclerotic homeostasis. But under sustained or severe pathological stress, dysregulated autophagy turns destructive, worsening vascular dysfunction and driving plaque progression and instability.

#### 3.1.7. Crosstalk Between Different Programmed Cell Death in AS

During the progression of disease, there is crosstalk between different programmed cell deaths; blocking one can cause cells to switch to another. For example, the use of caspase inhibitors can prevent apoptosis and promote necrotic cell death; conversely, the activation of Caspase-8 promotes apoptosis whilst cleaving RIPK1 and RIPK3, thereby blocking the initiation of necrotic cell death [127]. During the execution phase of necrotic apoptosis, MLKL oligomerizes to form necrosomes, which translocate to the cell membrane to form pores, whilst simultaneously activating the NLRP3 inflammasome, thereby promoting the initiation of pyroptosis [128]. Furthermore, ox-LDL increases the expression of the RIPK3 and MLKL genes, leading to the occurrence of necrotic apoptosis, whilst also inducing the formation of the NLRP3 inflammasome, thereby triggering pyroptosis [129,130]. Autophagy initially suppresses apoptosis (by EVA-1 homolog A(EVA1A)-driven clearance of damaged mitochondria) and ferroptosis (by restoring GPX4/SLC7A11 and reducing lipid peroxidation) [131,132]. However, excessive autophagy paradoxically promotes death: NCOA4-dependent ferritinophagy liberates iron, fueling ferroptosis and inflammasome activation, while EVA1A overexpression causes autophagosome accumulation and caspase-3-mediated apoptosis [131,133].

In summary, there are links between different types of cell death, which can be regulated by various signaling pathways and environmental factors; this highlights the complexity of cell death. Understanding the interactions between different signaling pathways and the influence of the cellular environment on patterns of cell death is crucial for the development of therapies targeting these pathways.

### 3.2. PCD of ECs in MI

Myocardial infarction (MI), a leading global cause of death, is the primary cause of mortality in coronary heart disease. Recent single-cell multi-omics and genetic lineage-tracing studies show that ECs undergo transient metabolic, mesenchymal, hematopoietic, and pro-inflammatory phenotypic shifts in the damaged microenvironment [134]. Approximately 25% of non-myocytes are ECs, which play key roles in vascular repair, inflammation regulation, and cardiac function maintenance [135]. These studies confirmed the crucial role of ECs in MI, highlighting the need to further investigate the effect and molecular mechanism of PCD of ECs.

#### 3.2.1. Apoptosis of ECs in MI

Ischemia and hypoxia impair mitochondrial oxidative phosphorylation in ECs, causing ATP depletion. This energy crisis triggers the apoptosis of ECs, with pathological changes including abnormal mPTP opening, loss of mitochondrial membrane potential, calcium overload, and dysregulated mitochondrial fission and autophagy [136]. RNA-seq analysis revealed extrinsic pathway activation leading to EC apoptosis post-MI. Thirteen extrinsic pathway-associated genes were substantially upregulated, including thrombospondin-1 (THBS1), plasminogen activator inhibitor-1 (SERPINE1), secretogranin II (SCG2), telomerase reverse transcriptase (TERT), Fas ligand (FASL), bone morphogenetic protein 4 (BMP4), ICAM1, and tumor necrosis factor alpha-induced protein 3 (TNFAIP3). These genes likely drive classical death receptor signaling (e.g., Fas/FasL and TNF-α/TNFR1), leading to caspase-8 activation and apoptosis [137]. Endogenous-pathway-related genes X-box binding protein 1 (XBP1) and NFE2L2 were overexpressed, along with three genes involved in both endogenous and exogenous signaling: TNF, MAPK7, and homeodomain-interacting protein kinase 1 (HIPK1) [138].

Mechanistically, the PI3K/AKT pathway critically regulates EC apoptosis post-MI [139]. miR-124 promotes EC apoptosis and inhibits proliferation by activating p38 MAPK and suppressing PI3K/AKT, potentially contributing to vascular endothelial injury in MI [139]. On the other hand, serum from ST-segment elevation myocardial infarction (STEMI) patients induced significant endothelial injury in vitro, potentially via pro-apoptotic factors such as proprotein convertase subtilisin/kexin type 9 (PCSK9) [140]. In summary, apoptosis of ECs is triggered by ischemia–hypoxia and the ensuing oxidative stress, and is executed mainly via the death receptor and mitochondrial pathways. Transcriptome has not only validated classical pathway activation but also uncovered novel regulatory genes, significantly expanding the understanding of the extrinsic apoptotic pathway. These findings provide a crucial theoretical basis for protecting endothelial function and improving MI prognosis via multi-target interventions.

#### 3.2.2. Pyroptosis of ECs in MI

Evidence shows that plasma levels of NLRP3 and caspase-1 are markedly elevated in MI patients [141]. In vitro studies show that NLRP3 is expressed in cardiac microvascular endothelial cells (CMECs) but nearly absent in cardiomyocytes [142]. In coronary artery disease, hyperglycemia, and hyperhomocysteinemia, oxidative stress activates the NLRP3 inflammasome, triggering caspase-1–mediated GSDMD cleavage and pyroptosis of ECs, which promotes MI [143,144,145]. Concurrently, the pyroptosis of ECs contributes to MI pathology by promoting microvascular obstruction (MVO) and myocardial hemorrhage, thereby impairing myocardial remodeling and functional recovery [146]. In the case of SARS-CoV-2 infection, an infection- or immune-driven cytokine storm can trigger EC pyroptosis, which underlies cardiovascular complications such as arrhythmias, stroke, and MI [147]. Pyroptosis of ECs is regulated by non-coding RNAs, transcription factors such as interferon regulatory factor 1 (IRF1), and enzymatic modifications such as HDAC11-mediated ERG acetylation, which modulate the NLRP3 inflammasome and associated signaling pathways [148,149]. Not only that, but ROS drive pivotal catalysts for EC pyroptosis [150,151]. In summary, CMECs are the primary site of NLRP3 inflammasome expression and activation, serving as a critical hub for tissue injury. Targeting key regulators of EC pyroptosis offers a promising novel therapeutic strategy.

#### 3.2.3. Necroptosis of ECs in MI

Clinical studies show elevated plasma RIPK3 in MI patients, correlating with higher risk of adverse cardiovascular events. It is important to note that apoptosis preserves plasma membrane integrity, manifesting only as zeiosis, and thus elicits minimal inflammatory factor release. In contrast, necroptosis involves plasma membrane rupture, recruiting diverse immune cells and driving robust, destructive inflammation. This gives it a dual role in post-MI myocardial inflammation and remodeling. On one hand, it releases cellular contents to activate immune responses and amplify inflammation; on the other, excessive inflammation can exacerbate necroptosis, creating a vicious cycle that worsens myocardial injury [152]. In summary, necroptosis of ECs exacerbates post-MI inflammation and cardiac remodeling. The core necroptotic RIPK1/RIPK3/MLKL pathway drives pathologically pro-inflammatory effects in MI. Thus, inhibiting this pathway to ameliorate endothelial dysfunction and restore cardiac function is the most promising therapeutic strategy.

#### 3.2.4. Ferroptosis of ECs in MI

During MI, local ischemia/hypoxia elevates oxidative stress via iron-catalyzed Fenton reactions, generating ROS that drive lipid peroxidation, thereby causing ferroptosis of ECs [35,153]. Ferroptosis of ECs increases vascular permeability and inflammatory factor release, exacerbating endothelial injury and impairing blood flow in ischemic myocardium, thereby accelerating disease progression [154]. Hence, it has reported that ferroptosis-related gene expression levels predict post-MI heart failure and other adverse events, enabling risk stratification [155].

Under post-MI hypoxic stress, ECs ferroptosis is marked by ROS and lipid peroxide accumulation and GPX4 downregulation. Bulk RNA-seq revealed significant upregulation of six ferroptosis-related genes in EC following MI. scRNA-seq further showed significant upregulation of ferroptosis-related genes in both ECs and stromal cells, implicating EC ferroptosis in MI pathogenesis [156]. However, some genes inhibit ECs ferroptosis by regulating GPX4. For instance, ovarian tumor domain protease 5 (OTUD5) suppresses EC ferroptosis under hypoxia by activating NF-κB/p65 signaling—reducing ROS and lipid peroxidation, restoring GPX4 expression, and downregulating ACSL4—thereby preserving pro-angiogenic function [157]. Additionally, activation of aldehyde dehydrogenase 2 (ALDH2) relieves myocardial infarction in a mouse model of MI through inhibiting EC ferroptosis, and promoting their proliferation and migration (Figure 2) [86]. In conclusion, current therapeutic research focuses on GPX4 and its associated regulatory genes. However, iron chelators and lipid peroxidation inhibitors such as ferrostatin-1, which can effectively inhibit cardiomyocyte ferroptosis, reduce myocardial injury, and improve cardiac function, have rarely been studied in ECs related to MI, warranting more attention.

#### 3.2.5. Autophagy of ECs in MI

During MI, moderate autophagy in ECs protects against ischemia-induced apoptosis by clearing damaged components and maintaining metabolic homeostasis [158,159]. On the other side, prolonged or excessive autophagy in ECs can trigger autophagic cell death, worsening vascular dysfunction and injury [160,161]. Under ischemic stress, AMPK activation generally promotes autophagy in ECs [162]. It is also significant to note that other regulatory factors, such as miR-92a-3p, enhance EC autophagy by upregulating autophagy-related gene 4a (ATG4a), while miR-106a suppresses autophagy and angiogenesis by targeting and inhibiting ATG7 [163,164]. Angiogenic factor 1 (AGGF1) shows therapeutic potential for coronary artery disease. In mice with myocardial infarction (MI), AGGF1 protein therapy stimulated therapeutic angiogenesis, leading to improved survival, increased ejection fraction, reduced infarct size, attenuated cardiac apoptosis and fibrosis, and robust recovery of myocardial function and contractility. AGGF1 can activates JNK in ECs, leading to autophagy and the formation of the Becn1-Vps34-ATG14 complex in MI (Figure 3) (Table 1) [93]. Autophagy is a dynamic, complex process, the dysregulation of which can cause cell death and tissue damage. Although EC autophagy targeting offers a novel strategy for MI treatment, its clinical translation remains challenging.

### 3.3. PCD of ECs in Myocardial I/R Injury

Myocardial I/R injury is a major clinical challenge in MI, significantly affecting patient prognosis. Although PCI restores coronary flow, reperfusion itself can worsen cardiomyocyte death and dysfunction, and current interventions remain limited in efficacy [165,166]. Myocardial I/R injury not only damages cardiomyocytes but also significantly affects biological functions in ECs [167,168]. PCD of ECs trigger inflammatory infiltration, microthrombi, and microvascular dysfunction, culminating in the “no-reflow” phenomenon, thereby worsening myocardial injury [169,170,171].

#### 3.3.1. Apoptosis of ECs in Myocardial I/R Injury

In the early phase of reperfusion, cell death initially manifests in the ECs of small coronary vessels. The radial spread of cell death to surrounding cardiomyocytes indicates that reperfusion induces ECs to release soluble pro-apoptotic mediators, thereby promoting cardiomyocyte apoptosis [172]. Simultaneously, the resupply of oxygen exacerbates ROS production in ECs, further activating the pro-apoptotic protein Bax while inhibiting the anti-apoptotic protein Bcl-2, ultimately leading to decreased mitochondrial membrane potential and apoptosis [136,173]. Improving mitochondrial dysfunction during this process has emerged as a potential therapeutic strategy for I/R injury. Among the marketed drugs with proven cardioprotective effects, empagliflozin improves mitochondrial function and reduces EC apoptosis and microvascular dysfunction by inhibiting the dephosphorylation of mitochondrial fission protein 1 (Fis1) to block mitochondrial fission [174]. As a potassium channel opener, pinacidil reduces mitochondrial calcium influx and damage by overexpressing calreticulin (CRT) to inhibit inositol 1,4,5-trisphosphate receptors (IP3Rs) and mitochondrial calcium uniporter (MCU), thereby alleviating calcium overload and mitochondrial-dependent EC apoptosis, which improves perfusion and reduces infarct size [175].

In myocardial I/R jury, multiple pathways are involved in EC apoptosis. Activation of the PI3K/Akt pathway protects ECs. Sufentanil alleviates OGD/R-induced barrier dysfunction in human CMECs via PI3K/Akt activation, and suppresses apoptosis [176]. Shexiang Baoxin Pill (SBP) alleviates EC apoptosis, oxidative stress, and inflammation after myocardial I/R injury by upregulating S1PR1 to activate the downstream AKT pathway and promote vascular regeneration, thereby improving cardiac function [177]. After myocardial I/R injury, aberrant MAPKK4/p38 signaling exacerbates injury and apoptosis in CMECs. The active components of Buyang Huanwu Decoction (BYHW), astragaloside IV and ligustrazine can alleviate EC apoptosis and microvascular dysfunction by modulating the MAPKK4/p38 pathway [178]. The activation of the transient receptor potential vanilloid 4 (TRPV4) channel increases cardiac EC permeability and promotes apoptosis via the PKC/RhoA/MLC pathway, worsening vascular hyperpermeability and myocardial injury after reperfusion. Further studies show that selective TRPV4 antagonists significantly reduce infarct size and preserve cardiac function [179].

Studies show shear stress reverses CMEC apoptosis via the YAP/miR-206/PDCD4 pathway, alleviating microvascular I/R injury [180]. In cardiac I/R injury, ischemia causes lactate accumulation and tissue acidosis. At physiological pH (7.4), caspase-8 inhibition fails to block TNF-α–induced apoptosis in mouse cardiac VECs but instead triggers necroptosis. In contrast, at acidic pH (6.5), TNF-α–induced death displays apoptotic morphology and is sensitive to caspase-8 inhibition. Acidosis promotes phosphorylation of RIPK1, RIPK3, and MLKL, but their activation is self-limiting—and also enhances AIF cleavage and nuclear translocation. Thus, microenvironmental pH dictates the mode of cell death by modulating apoptosis and necroptosis signaling [181]. Granzyme B (GZMB) is upregulated in MI patients and identified as a key gene by bioinformatics analysis. miR-518a-5p reduces hypoxia-reoxygenation injury by targeting GZMB [182]. CircZNF609 exacerbates myocardial I/R injury by promoting cardiomyocyte and HUVEC apoptosis via the miR-214-3p/prostaglandin-endoperoxide synthase 2 (PTGS2) axis [183]. In summary, in myocardial I/R injury, EC injury mediates microvascular dysfunction and myocardial damage. Mitochondrial dysfunction is central to EC apoptosis; thus, targeting mitochondrial function is crucial for EC protection.

#### 3.3.2. Pyroptosis of ECs in Myocardial I/R Injury

Myocardial I/R induces significant pyroptosis in CMECs, marked by upregulation of NLRP3, caspase-1, and GSDMD, and release of IL-1β and IL-18, leading to microvascular dysfunction [184]. The triggers of EC pyroptosis by I/R-induced are mainly oxidative stress, calcium overload, and mitochondrial damage. Among these, ROS from oxidative stress are key activators of the NLRP3 inflammasome and EC pyroptosis, highlighting redox status as central to pyroptosis regulation [150,185].

The regulatory mechanism of EC pyroptosis in myocardial I/R injury involves multiple factors and is mediated through the NLRP3/Caspase-1/GSDMD pathway. Under hypoxia-reoxygenation conditions, the long non-coding RNA NEAT1 regulates the NLRP3 inflammasome via the miR-204/BRCC3 axis, thereby influencing EC pyroptosis [186]. Meanwhile, under OGD, upregulated HSP90 stabilizes EZH2, which suppresses miR-22 via H3K27me3 modification. Reduced miR-22 derepresses NLRP3, triggering Caspase-1 activation, GSDMD cleavage, and IL-1β/IL-18 release, leading to EC pyroptosis [187]. Ubiquitin specific peptidase 5 (USP5) stabilizes RIPK1 by removing its K63-linked polyubiquitin chains, thereby activating caspase-8 and cleaving GSDMD to induce pyroptosis in myocardial microvascular ECs (Figure 1) [80]. Elevated levels of high-mobility group box 1 (HMGB1) in both the heart and circulation following I/R was detected in a mouse model, with a portion originating from cardiac vascular endothelial cells and cardiomyocytes. Moreover, endothelial HMGB1 promotes EC pyroptosis via AIM2 inflammasome activation, exacerbating myocardial I/R injury in in vivo study (Figure 1) (Table 1) [81]. In addition, beclin-1 upregulation enhances autophagic flux and inhibits caspase-4 activation and GSDMD cleavage [188]. In summary, EC pyroptosis critically contributes to microcirculatory dysfunction in myocardial IR injury. Targeting key signaling molecules (NLRP3/Caspase-1/GSDMD) holds promise for the clinical prevention and treatment of myocardial IR injury.

#### 3.3.3. Necroptosis of ECs in Myocardial I/R Injury

In myocardial I/R injury, the necroptosis of CMECs critically exacerbates microcirculatory dysfunction, primarily driven by upregulated RIPK1 and RIPK3 expression and activity. This triggers MLKL phosphorylation and membrane translocation, causing EC death. Necroptosis directly impairs microvessel integrity and worsens myocardial injury and dysfunction by releasing inflammatory mediators that promote local immune cell infiltration [189,190]. Moreover, MLKL-dependent EC necroptosis triggers erythrocyte hemolysis and microvascular occlusion, exacerbating myocardial I/R injury [191]. In addition to the classical RIPK1/RIPK3/MLKL axis, mitochondrial dysfunction critically regulates necroptosis. The regulatory protein of the mitochondrial permeability transition pore (mPTP), cyclosporine D (CypD), promotes apoptosis-inducing factor (AIF) translocation to the nucleus, causing DNA fragmentation and accelerating EC necroptosis [189]. Further studies show that Cu^2+^ released during myocardial I/R synergizes with homocysteine (Hcy) to induce necroptosis in CMECs. This occurs via cooperative activation of NOX and eNOS by Cu^2+^ and Hcy, driving excessive ROS and NO production, which combine to form cytotoxic peroxynitrite (ONOO^−^) and trigger necroptosis [192]. In summary, the inhibition of EC necroptosis has emerged as a potential therapeutic strategy. Blocking the classical RIPK1/RIPK3/MLKL axis or inhibiting CypD function can significantly reduce endothelial necroptosis, protect the microvasculature, and dampen inflammation [82,189]. Moreover, Sarco/endoplasmic reticulum Ca^2+^-ATPase (SERCA) overexpression protects microvessels from I/R injury by maintaining calcium homeostasis, preventing mitochondrial calcium overload, and inhibiting aberrant mPTP opening [193].

#### 3.3.4. Ferroptosis of ECs in Myocardial I/R Injury

Ferroptosis, an iron-dependent, regulated cell death driven by lipid peroxide accumulation, plays a key role in the pathology of myocardial I/R injury [194]. Notably, ferroptosis in CMECs is particularly crucial, characterized by GSH depletion, GPX4 inhibition, and lipid peroxide accumulation [37,194]. Ferroptosis of ECs directly disrupts microvascular integrity, increasing permeability and impairing perfusion, thereby driving the “no-reflow” phenomenon and secondary myocardial injury during I/R [88]. Recent research has identified key molecular pathways regulating EC ferroptosis, primarily targeting antioxidant balance. The SLC7A11/GPX4 axis is the canonical anti-ferroptosis pathway. SLC7A11, a core component of system Xc^−^, mediates cystine uptake for GSH synthesis, thereby sustaining GPX4 activity to clear lipid peroxides; however, during myocardial I/R, this pathway is frequently suppressed [194]. ECs show reduced expression of soluble guanylate cyclase soluble subunit alpha 1 (GUCY1A1) in myocardial I/R. In the acute phase of ischemia–reperfusion injury, EC-specific GUCY1A1 knockout mice further impaired microvascular perfusion, expanded the no-reflow area, and increased infarct size. These changes subsequently worsened cardiac dysfunction and adverse structural remodeling during the chronic phase. However, activated GUCY1A1 inhibits EC ferroptosis though phosphorylating LDHA, thus leading to GPX4 phosphorylation and stabilization, thereby relieving myocardial I/R injury (Figure 2) (Table 1) [88]. On the other hand, the Nrf2/HO-1 pathway, an essential antioxidant defense mechanism, suppresses ferroptosis upon activation. Pigment-epithelium-derived factor (PEDF) and its active 34-mer peptide inhibit hypoxia-reoxygenation-induced endothelial cell ferroptosis by activating this pathway. Recent studies show that iron chelators, lipid peroxidation inhibitors, and Nrf2 pathway activators effectively suppress EC ferroptosis, alleviating myocardial I/R injury and improving cardiac function [195]. However, the specific regulatory role of ferroptosis in endothelial cells within myocardial I/R injury remains to be further clarified.

#### 3.3.5. Autophagy of ECs in Myocardial I/R Injury

Dysfunctional autophagy, a key intracellular degradation process, is closely linked to microcirculatory disturbances and the no-reflow phenomenon [196]. I/R severely damages ECs and disrupts intracellular homeostasis in the myocardium. Autophagy in ECs plays a dual role in I/R injury: (1) appropriate autophagy promotes survival by clearing damaged organelles, and (2) excessive activation worsens damage [197,198,199]. Furthermore, autophagy in ECs exhibits distinct spatiotemporal specificity during I/R. During the early ischemic phase, autophagic activity is upregulated and exerts protective effects primarily through the clearance of damaged mitochondria. At this stage, expression levels of key autophagy-related proteins including Beclin1 and the lipidated form of LC3 (LC3-II) are significantly increased, while p62 (SQSTM1) degradation is enhanced. Evidence shows that elevated Beclin1 overexpression improves survival, reduces infarct size, and attenuates the no-reflow area [188]. However, during reperfusion, ROS accumulation can overactivate autophagy via the NF-κB p65–Beclin1 pathway, triggering autophagic cell death [198,199,200]. Moreover, excessive autophagy impairs microvascular barrier function by disrupting endothelial junction proteins like VE-cadherin [196]. During this process, iNOS also promotes EC migration and apoptosis by upregulating autophagy ATG5 and LC3B, whereas its inhibitor L-NAME reverses these effects [197].

Mechanistically, the AMPK/mTOR pathway centrally regulates autophagy in endothelial cells. Melatonin inhibits AMPK/mTOR-dependent autophagy, whereas the AMPK activator AICAR can counteract this effect [201]. Exosomal lncRNA LINC00174 alleviates myocardial injury by modulating Akt/AMPK signaling to suppress autophagy and apoptosis [202]. Dexamethasone (Dex) activates the Peroxisome Proliferator-Activated Receptor Delta (PPARδ)–AMPK–Peroxisome Gamma Coactivator-1 Alpha (PGC-1α) pathway in CMECs, suppressing OGD/R-induced excessive apoptosis [203]. In addition, the restoration of autophagy flux (including lysosomal autophagy function) enhances microvascular function [204].

## 4. Cell and Gene Therapy for Protecting ECs Function in IHD

### 4.1. Cell and Gene Therapy for Protecting ECs Function in AS

In the field of stem cell therapy for AS, mesenchymal stem cells (MSCs) and endothelial progenitor cells (EPCs) have emerged as two cell types of particular therapeutic significance. Their potential to advance novel treatment strategies for AS is underpinned by anti-apoptotic effects and the capacity to promote endothelial repair [205]. MSCs exert therapeutic effects through multiple mechanisms, involving regulating lipid metabolism, suppressing inflammation, promoting endothelial repair, and stabilizing plaques. Their secreted extracellular vesicles (EVs)—especially exosomes—act as natural nanoscale carriers that deliver bioactive molecules (such as proteins, mRNAs, and non-coding RNAs) precisely to target cells. Evidence has shown that MSC-derived exosomes inhibit EC migration and plaque formation via miR-145, or protect ECs from oxidative stress-induced apoptosis via miR-342-5p [206,207]. EPCs, as precursors of mature ECs, serve as biomarkers of vascular health, with their number and functional capacity reflecting the integrity and homeostatic status of the vasculature. Upon vascular injury, EPCs are mobilized from bone marrow niches and are home to sites of damage, where they contribute to neovascularization and endothelial repair [208]. Extracellular vesicles released by EPCs have also been demonstrated to play a crucial role in vascular repair, which alleviate AS though inhibiting ECs ferroptosis by microRNA-199a-3p delivery [209,210].

Gene therapy for atherosclerosis primarily encompasses two strategies: systemic gene therapy aimed at modulating lipid metabolism, and local gene therapy directed specifically at vascular lesions [211]. The CRISPR-Cas9 system enables precise, efficient, and persistent modification of genes implicated in EC function. In AS, dysregulated transforming growth factor-beta (TGF-β) contributes to endothelial dysfunction, while targeted editing of TGF-β in ECs exerts the effect of protecting endothelial function and alleviating AS [212].

### 4.2. Cell and Gene Therapy for Protecting EC Function in MI and Myocardial I/RI

EVs carry bioactive molecules including microRNAs and proteins as stem cell paracrine effects to precisely regulate ECs’ survival and function [213]. In MI, EVs from cardiac mesenchymal stem cells (C-MSCs) overexpressing the Notch1 intracellular domain (N1ICD) were enriched with pro-repair proteins (LOXL2 and biglycan), which inhibi apoptosis in both ECs and cardiomyocytes [214]. MSC-derived exosomes overexpressing miR-214 (miR-214OE-Exo) promote endothelial cell function in vitro and in vivo by targeting phosphatase and tensin homolog (PTEN) and activating p-AKT signaling, thereby enhancing myocardial repair [215]. miR-125a-5p, found in the MSC-derived exosome, and its mimic (agomir), regulate EC function, thereby reducing cardiomyocyte apoptosis and inflammation and improving cardiac function [216]. EVs secreted by adipose-derived stem cells (ADSCs) after hypoxic preconditioning (ADSC-EVs[H]) have been shown to regulate the proliferation, oxidative stress, and apoptosis in hypoxia/reoxygenation-induced human dermal microvascular endothelial cells (HDMECs) [217]. In addition to exosome delivery strategies, it has been confirmed that multiple miRNAs directly participate in apoptosis following I/R injury [218]. miR-27b targets FOXO1 in ECs to inhibit the Akt/FOXO1 pathway, thereby counteracting TNF-α-induced EC apoptosis [219].

Mitochondrial transplantation technology, as a developing cell therapy strategy, seeks to enhance energy metabolism in ECs following myocardial I/R injury by directly supplementing functional mitochondria. Despite their low mitochondrial content, CMECs suffer significant mitochondrial oxidative stress and dynamic dysfunction during I/R injury, leading to cellular dysfunction and apoptosis, potentially even before cardiomyocyte damage occurs [173].

A primary strategy for improving clinical outcomes in MI and I/R injury is to enhance EC repair capacity and pro-angiogenic function. The vascular endothelial growth factor (VEGF) family has been identified as the primary target for promoting myocardial angiogenesis, comprising VEGF-A, VEGF-B, VEGF-C, VEGF-D, and placental growth factor [220]. In models of porcine MI, adenoviral vector-mediated delivery of the VEGF-D gene has been shown to safely and effectively induce angiogenesis, improving blood perfusion and cardiac function [221]. BMP6 regulates EC function by modulating VEGFR2 and the Hippo pathway effector TAZ [222]. Activation of the Notch signaling pathway through Elabela (ELA) gene therapy by delivering the ligand Jagged1 can synergistically enhance the VEGF pathway, significantly promote angiogenesis and inhibit fibrosis [223,224]. Overexpression of MEIS1 can also induce multiple pro-angiogenic factors, markedly increasing capillary density [225]. These strategies aim to induce EC growth, rapidly repair the microvascular barrier, and reduce no-reflow and secondary myocardial necrosis.

### 4.3. Limitations and Future Directions in Cell and Gene Therapy

Despite encouraging preclinical results, the clinical translation of cell and gene therapy still faces major hurdles: transplanted stem cells exhibit poor survival and limited homing, with very few cells reaching the target tissue while the majority are trapped in the liver, spleen, and lungs. Even stem cells that are home to and engraft within infarcted myocardium have shown no evidence of giving rise to new cardiac cells [226]. Gene therapy aimed at improving the local microenvironment may therefore represent a more effective strategy to enhance the survival and differentiation of transplanted cells. Furthermore, ensuring high specificity and minimizing off-target edits remain challenging in clinical settings [227,228]. In particular, the potential off-target effects and immunogenicity of CRISPR-Cas9, together with the tumorigenic risk after stem cell transplantation, demand rigorous long-term evaluation and more refined regulatory control [229]. The complex heterogeneity of coronary heart disease (CHD) and the incomplete understanding of its pathogenic mechanisms further hinder the broad adoption of cell and gene therapy. Future directions emphasize integrating multi-omics profiling, synthetic biology, and AI-aided biomaterials to develop therapeutics [230]. For example, patients with atherosclerosis differ considerably in genetic background, disease stage, and microenvironment. Common mutations affect proprotein convertase subtilisin/kexin type 9 (PCSK9) and the low-density lipoprotein receptor (LDLR) gene; however, certain loss-of-function mutations in PCSK9 lead to persistently low plasma LDL levels and confer a lower risk of cardiovascular disease [231]. These observations highlight the need to advance toward individualized and regulatable precision medicine.

## 5. Perspective

In IHD, the PCD of ECs has expanded from a single focus on apoptosis to a complex regulatory network encompassing multiple forms of cell death, including pyroptosis, necroptosis, ferroptosis, autophagy, ferroptosis, panoptosis, and so on. Under stress conditions, multiple PCD pathways in ECs do not operate in isolation. Rather, they converge on shared hub molecules, thereby forming a highly coordinated and intricately interconnected regulatory network. This network plays a continuous yet distinct role in AS, MI, and I/R injury. Thus, identifying modality-specific PCD markers will improve disease staging, risk assessment, and therapeutic monitoring. The crosstalk among these death pathways suggests combined interventions may be more effective. Moreover, research on emerging cell death modalities such as cuproptosis and parthanatos in IHD is still in its infancy, with their exact pathological contributions and regulatory mechanisms yet to be elucidated.

Cell therapy protects ECs by improving the local microenvironment, whereas gene therapy precisely reprograms endothelial function at the molecular level. Therapeutic priorities differ across diseases: in AS, the focus is on EC protection via risk-factor reduction (e.g., hyperlipidaemia); in MI and I/R injury, rapid vascular repair and inflammation modulation are prioritized. Despite promising preclinical results, efficiently and selectively delivering therapeutics to lesion sites without affecting healthy tissue remains a major challenge. Moreover, off-target gene editing, immunogenicity, and tumorigenicity risks with stem cell transplantation demand more rigorous long-term evaluation and refined regulatory oversight.

## 6. Conclusions

Here, we systematically examine the major forms of PCD of ECs in IHD, with emphasis on their distinct molecular activation mechanisms and regulatory networks. From this perspective, PCD of ECs in IHD is not a monolithic process but rather a dynamically interlinked and context-dependent cascade, wherein these pathways exhibit extensive crosstalk and functional redundancy. Nevertheless, emerging therapeutic strategies aimed at restoring endothelial integrity and function and based therapies and targeted gene interventions hold significant promise. These approaches may offer novel, mechanism-driven avenues for the clinical management of IHD.

## Figures and Tables

**Table 1:** Regulatory factor or pathway of ECs’ PCD in IHD.

Regulatory Factor or Pathway	Programmed Cell Death	Disease	In Vitro/Vivo	Potential Regulatory Mechanisms	Reference
PERK	Apoptosis	AS	In vitro and vivo	Activating CHOP	[69]
MBOAT2	Pyroptosis	AS	In vitro and vivo	Activating NLRP3 inflammasome	[76]
IKKε/STAT1	Pyroptosis	AS	In vitro and vivo	Activating NLRP3 inflammasome	[77]
Rnd3/TRAF6	Pyroptosis	AS	In vitro and vivo	Activating the NF-κB/NLRP3 axis	[78]
CCL14	Pyroptosis	AS	In vitro and vivo	Activating the NF-κB/NLRP3 axis	[79]
USP5	Pyroptosis	MI/RI	In vitro and vivo	Stabilizes RIPK1	[80]
HMGB1	Pyroptosis	MI/RI	In vitro and vivo	Activating AIM2 inflammasome	[81]
AQP1	Necroptosis	AS	In vitro and vivo	Suppress RIPK3 and MLKL expression	[82]
CTRP13	Ferroptosis	AS	In vitro and vivo	Activating GCH1/BH4 Inhibiting KLF4 expression	[83,84]
DDHD2	Ferroptosis	AS	In vitro and vivo	Activating Nrf2/GPX4 pathway	[85]
ALDH2	Ferroptosis	MI	In vitro and vivo	Activating xCT	[86]
FABP3	Ferroptosis	AS	In vitro	Anhibiting GPX4	[87]
GUCY1A1	Ferroptosis	MI/RI	In vitro and vivo	Activating LDHA/GPX4	[88]
P38	Ferroptosis	MI	In vitro and vivo	Activating NCOA4	[89]
SESN1	Autophagy	AS	In vitro	Activating AMPK	[90]
ARG2	Autophagy	AS	In vitro and vivo	Activating AMPK	[91]
CAV1	Autophagy	AS	In vitro and vivo	Suppress ATG5-ATG12 complex	[92]
AGGF1	Autophagy	MI	In vitro and vivo	Promote Becn1-Vps34-ATG14 complex	[93]

The table summarizes the regulatory factor or pathway of programmed cell death in endothelial cells and potential regulatory mechanisms during IHD progression.

## Data Availability

No new data were created or analyzed in this study.

## Abbreviations

The following abbreviations are used in this manuscript:

IHD	Ischemic heart disease
AS	Atherosclerosis
MI	Myocardial infarction
I/R	Ischemia/reperfusion
PCD	Programmed cell death
ECs	Endothelial cells
Apaf-1	Apoptotic protease-activating factor-1
ATP	Adenosine triphosphate
Nrf2	Nuclear factor erythroid 2-related factor 2
HO-1	Heme oxygenase-1
ROS	Reactive oxygen species
ER	Endoplasmic reticulum
TNFR	Tumor necrosis factor receptor
DISC	Death-inducing signaling complex
GSDMD	Gasdermin D
NT	N-terminal fragment
IL	Interleukin
LPS	Lipopolysaccharide
RIPK	Receptor-interacting protein kinase
MLKL	Mixed-lineage kinase domain-like protein
DAMPs	Damage-associated molecular patterns
ZBP1	Z-DNA binding protein 1
NLRP	Nod-like receptor protein
AIM2	Absent in melanoma 2
FADD	Fas-associated death domain
ASC	Apoptosis-associated speck-like protein containing a CARD
PUFA	Polyunsaturated fatty acid
TfR1	Transferrin receptor 1
GPX4	Glutathione peroxidase 4
CMA	Chaperone-mediated autophagy
mTOR	Mammalian target of rapamycin
mTORC1	Mammalian target of rapamycin complex 1
ULK1	UNC-51-like autophagy receptor kinase 1
PI3K	Phosphatidylinositol 3-kinase
PI3P	Phosphatidylinositol-3-phosphate
LC3-PE	Microtubule-associated protein 1 light chain 3-phosphatidylethanolamine
AMPK	AMP-activated protein kinase
MAPK	Mitogen-activated protein kinase
JNK	c-Jun N-terminal kinase
miRNAs	MicroRNAs
PARP1	Poly(ADP-ribose) polymerase 1
LDL	Low-density lipoprotein
ox-LDL	Oxidized low-density lipoprotein
BAX	BCL-2-associated X protein
BCL-2	B-cell lymphoma 2
NF-κB	Nuclear factor kappa-B
JAK/STAT	Janus kinase/signal transducer and activator of transcription
GRP78	Glucose-regulated protein 78
CHOP	C/EBP homologous protein
PERK	Protein kinase R-like endoplasmic reticulum kinase
IRE1α	Inositol-requiring enzyme 1α
VCAM-1	Vascular cell adhesion molecule-1
ICAM-1	Intercellular adhesion molecule-1
ERα/ERβ	Estrogen receptor alpha/beta
NLRP3	NLR family pyrin domain containing 3
STAT1	Signal transducer and activator of transcription 1
TMAO	Trimethylamine N-oxide
MBOAT2	Membrane bound O-acyltransferase domain containing 2
SDHB	Succinate dehydrogenase complex iron sulfur subunit B
Rnd3	Rho family GTPase 3
TRAF6	TNF receptor associated factor 6
AQP1	Aquaporin-1
Hcy	Homocysteine
FABP3	Fatty acid-binding protein 3
CD36	Cluster of differentiation 36
GCH1/BH4	GTP cyclohydrolase I/tetrahydrobiopterin
CTRP13	C1q/TNF-related protein 13
KLF4	Krüppel-like factor 4
RAP1B	Ras-related protein 1B
OSS	Oscillatory shear stress
SESN1	Sestrin 1
ARG2	Arginase 2
RICTOR	Rapamycin-insensitive companion of mTOR
AKT	Protein kinase B
CTRP9	C1q/TNF-related protein 9
CuONPs	Copper oxide nanoparticles
TTM	Tetrathiomolybdate
NOX	NADPH oxidase
eNOS	Endothelial nitric oxide synthase
NAD^+^	Nicotinamide adenine dinucleotide
CMECs	Cardiac microvascular endothelial cells
MVO	Microvascular obstruction
IRF1	Interferon regulatory factor 1
HDAC11	Histone deacetylase 11
ERG	ETS-related gene
Fis1	Mitochondrial fission protein 1
CRT	Calreticulin
IP3Rs	Inositol 1,4,5-trisphosphate receptors
MCU	Mitochondrial calcium uniporter
TRPV4	Transient receptor potential vanilloid 4
PKC	Protein kinase C
RhoA	Ras homolog family member A
MLC	Myosin light chain
YAP	Yes-associated protein
PDCD4	Programmed cell death 4
GZMB	Granzyme B
PTGS2	Prostaglandin-endoperoxide synthase 2
NEAT1	Nuclear paraspeckle assembly transcript 1
BRCC3	BRCA1/BRCA2-containing complex subunit 3
HSP90	Heat shock protein 90
EZH2	Enhancer of zeste homolog 2
USP5	Ubiquitin specific peptidase 5
HMGB1	High mobility group box 1
CypD	Cyclosporin D
AIF	Apoptosis-inducing factor
SERCA	Sarco/endoplasmic reticulum Ca^2+^-ATPase
PEDF	Pigment epithelium-derived factor
MSCs	Mesenchymal stem cells
EPCs	Endothelial progenitor cells
TGF-β	Transforming growth factor-beta
EVs	Extracellular vesicles
C-MSCs	Cardiac mesenchymal stem cells
N1ICD	Notch1 intracellular domain
LOXL2	Lysyl oxidase-like 2
PTEN	Phosphatase and tensin homolog
ADSCs	Adipose-derived stem cells
HDMECs	Human dermal microvascular endothelial cells
FOXO1	Forkhead box O1
VEGF	Vascular endothelial growth factor
BMP6	Bone morphogenetic protein 6
VEGFR2	Vascular endothelial growth factor receptor 2
ELA	Elabela
MEIS1	Meis homeobox 1
